# Prognostic Factors and Clinical Features of Neonatal Splenic Rupture/Hemorrhage: Two Cases Reports and Literature Review

**DOI:** 10.3389/fped.2021.616247

**Published:** 2021-01-25

**Authors:** Han-Pi Chang, Ren-Huei Fu, Jainn-Jim Lin, Ming-Chou Chiang

**Affiliations:** ^1^Division of Neonatology, Department of Pediatrics, Chang Gung Memorial Hospital and Chang Gung University College of Medicine, Taoyuan, Taiwan; ^2^Division of Pediatric Critical Care Medicine, Department of Pediatrics, Chang Gung Memorial Hospital and Chang Gung University College of Medicine, Taoyuan, Taiwan; ^3^Graduate Institute of Clinical Medical Sciences, Chang Gung University College of Medicine, Taoyuan, Taiwan

**Keywords:** neonates, spleen, splenic hematoma, splenic hemorrhage, splenic laceration, splenic rupture

## Abstract

**Background:** Neonatal splenic rupture/hemorrhage (SRH), an extremely rare and potentially fatal presentation, can spontaneously resolve without surgical treatment; However, treatment approaches remain controversial. The present study aimed to describe and analyze the clinical features and therapies of neonatal SRH and therapeutic approaches.

**Methods:** We present the cases of two patients and review another 37 cases reported in English-literature. The literature search included all articles published in PUBMED from inception between January 1968 and December 2019. Demographic data, precipitating factors, clinical characteristics including presenting symptoms and signs, presenting time, age at SRH presentation, imaging findings, as well as treatments and outcomes were analyzed.

**Results:** In addition to the two cases treated at our hospital, 37 neonates with SRH were reported during the study period. The rate of full-term neonates was 72% (28/39). The cause was idiopathic in most cases, and congenital coagulation disorders were underlying causes in 13% (5/39) of the cases. The most common presenting symptom and sign of neonatal SRH were pallor or anemia, followed by abdominal discoloration/distension. Additionally, 18% (7/39) of the cases presented with scrotal hematoma or swelling. The age at SRH presentation ranged between 3 h and 5 days of age. Abdominal ultrasonography or computed tomography was used as the diagnostic tool. Twenty-seven cases (69%) received surgical management. The prognosis was comparable between the neonates treated with splenectomy and those treated with non-surgical approaches. The mortality rate was 18% (7/39) in the study cohort. SRH presentation at ≤12 h of age was associated with higher mortality compared to SRH presenting time at >12 h of age (odds ratio 25.0, 95% CI 2.514–248.575, *p* = 0.001).

**Conclusion:** Our literature review revealed that the mortality rate of neonatal SRH was 18% and that the mortality risk was higher in neonates presenting with SRH symptoms and signs at ≤12 h of age.

## Introduction

Intra-abdominal hemorrhage, especially the splenic hemorrhage, is a rare event in neonates (1). The incidence of splenic rupture or hemorrhage is unknown, reflecting its rarity (2). The initial symptoms of neonatal splenic rupture/hemorrhage (SRH) are non-specific which may lead to the delayed diagnosis in some cases. Despite the potential fatality, SRH can spontaneously resolve without surgical treatment. Appropriate treatment strategies for neonatal SRH remain controversial. Herein, we report the cases of two neonates with SRH and review the other cases of neonatal SRH in the English-language literature, with the aim to describe the clinical features of neonatal SRH, to summarize therapies and outcomes, and to elucidate risk factors for mortality in neonates presenting with this rare condition.

## Patients and Methods

### Case Reports

#### Case 1

A baby girl was delivered at term to a gravida 2, para 1 mother through cesarean section due to fetal distress and placental abruption. Her birth weight (BW) was 2,460 g, and the 1 and 5 min Apgar scores were 6 and 7, respectively. After birth, the patient was flaccid and cyanotic. She was intubated and immediately resuscitated and with transfer to our hospital due to perinatal asphyxia.

At arrival to our hospital, the patient was pale and lethargic. Her vital signs were as follows: rectal temperature 36°C, pulse rate, 100/min; respiration rate 50/min, and blood pressure, 30/10 mmHg. She was ventilated with high frequency oscillation ventilation. Inotropic agents, blood transfusion and fluid resuscitation were administered. Laboratory data indicated metabolic acidosis. Additional laboratory parameters as follows: hemoglobin (Hb), 8.8 g/dL; the white blood cell count (WBCs), 13.9 × 10^3^/uL, and platelet counts 190 × 10^3^/uL. The prothrombin time (PT) and activated partial thromboplastin times were prolonged. The fibrinogen and D-dimer were <50 mg/dl and >10,000 FEU ng/mL. The Coombs test was negative. Cranial ultrasonography did not reveal intracranial or intraventricular hemorrhage. Perinatal asphyxia associated with maternal placental abruption and stage II hypoxic ischemic encephalopathy (HIE) were suspected. Therapeutic hypothermia was commenced at 5 h of age and erythropoietin was used as an adjuvant therapy. Abdominal ultrasound to investigate the cause of neonatal anemia revealed bloody ascites and splenic rupture with hemorrhage, and abdominal distension was noted during the evaluation. Emergent exploratory laparotomy and splenectomy were performed on the 1 day-old patient. During the laparostomy, massive hemoperitoneum, and splenic laceration with active bleeding were noted. Despite treatment, severe acute kidney injury, pulmonary hypertension and severe intraventricular hemorrhage occurred and was administered palliative treatment because of the expected poor outcome and high mortality.

#### Case 2

This 2 day-old female infant was delivered at gestational age of 39 weeks to a gravida 1 para 1 mother through vaginal delivery with a BW of 3,340 gm at an outside hospital. Apgar scores were 8 and 9 at 1 and 5 min, respectively. The antenatal examination was unremarkable. However, she developed pallor at 2 days of age; tachycardia and decreased activity were observed as well. Laboratory examination revealed severe anemia.

Physical examination on arrival at our hospital revealed pale appearance. Her vital signs were as follows: rectal temperature of 36.3°C; pulse rate, 143/min; respiration rate 26/min and blood pressure, 76/54 mmHg. Laboratory examination revealed the following: Hb 5.4 g/dL; WBCs, 17.92 × 10^3^/uL, and platelet count 207 × 10^3^/uL. Abdominal ultrasonography revealed an enlarged spleen and a heterogenous mass (4.0 × 3.7 × 3.8 cm) in left upper abdomen; thus, splenic laceration with hematoma and bleeding was suspected. Abdominal computed tomography (CT) scan showed peri-splenic hematoma, ~3.5 × 3 cm in size, and hemoperitoneum indicating splenic laceration. After blood transfusion and fluid replace therapy, the patient stabilized and her follow-up hemoglobin level was 17.4 g/dL. She was discharged without any complications.

### Patient Population

We used the keywords “neonatal splenic rupture,” “neonatal splenic laceration,” “neonatal splenic hemorrhage,” and “neonatal splenic hematoma,” to search articles in the PUBMED databases and reviewed those published articles from their inception between January 1968 and December 2019. In addition to the two cases presented herein, we identified 37 cases of neonatal SRH in the English-language literature.

### Data Collection and Statistical Analysis

This study was approved by the ethics committee of Chang Gung Medical Hospital which waived the requirement for informed consent to collect anonymized data. Demographic data, precipitating factors, clinical characteristics including presenting symptoms and signs, time of onset, imaging findings, treatments, and outcomes were collected and analyzed. Preterm birth was defined as birth before 37 weeks of gestation. Low birth weight was defined as BW < 2,500 g. Age of SRH presentation was defined as the time of the emergence of first symptoms or signs after birth. Categorical variables were reported as absolute numbers and percentages and analyzed using chi-square or Fisher' s exact test. Continuous variables were analyzed using parametric or non-parametric tests as appropriate. Continuous variables were reported as means with standard deviation for parametric data and as medians with interquartile range for non-parametric data. Risk factors for mortality were analyzed by univariate logistic regression and presented as odds ratios (OR) with 95% confidence intervals (CI). A *p* < 0.05 was considered to indicate statistical significance. The IBM SPSS statistics package, version 21 (IBM Corp., Armonk, NY, USA) was used for all statistical analyses.

## Results

There were 39 neonates with the diagnosis of SRH during the study period. The demographic data and detailed information on these 39 cases are presented in Table 1. The rates of full-term and preterm neonates were 72% (28/39) and 18% (11/39), respectively. The median BW was 3,200 (2,470–3,620) g, and four neonates were large for gestational age (LGA, BW > 4,000 g). Additionally, 74% (29/39) of the patients were delivered through vaginal delivery whereas the remaining patients 26% (10/39) were delivered through cesarean section. The time of SRH presentation ranged between 3 h and 5 days of age. The precipitating factors included prolonged labor (*n* = 3), birth trauma (*n* = 2), breech (*n* = 1), wandering spleen (*n* = 1), assaulted before delivery (*n* = 1), post resuscitation (*n* = 5), congenital deficiency in coagulation factor VII deficiency (*n* = 1), erythroblastosis fetalis (*n* = 4), hemophilia A (*n* = 3), family history of hemophilia A (*n* = 1), and preterm neonate (*n* = 11). Therefore, the rate of congenital coagulation disorders (congenital coagulation factor VII and hemophilia A) was 13% in the current cohort.

**Table 1:** Clinical characteristics of neonates with splenic rupture.

**Year**	**Case**	**GA**	**BW (Kg)**	**Precipitating Factors**	**Labor**	**AS (1'→ 5')**	**Initial symptoms/signs**	**Presenting time**	**Hemogram**	**Diagnostic or image studies**	**Treatment**	**Outcome**
1968	1 (3)	Term	3.63	Perinatal asphyxia Birth trauma	VD	1→ 1	Grunting, pale, and shock	9 h	HCT 21	Paracentesis	Splenectomy	Alive
1968	2 (4)	Term	3.75	NA	VD	9→ 9	Tachypnea, tachycardia	43 h	Hb 7.0	X-ray	Splenectomy	Alive; normal
1971	3 (5)	Term	4.2	NA	VD	9→ 9	Swollen and blue scrotum, abdominal distension	6 h	Hb 8.0 PLT 130K	Intravenous pyelography	Splenectomy	Alive; normal follow-up examination
1973	4 (6)	36 weeks	1.7	Erythroblastosis fetalis	C/S	NA	Hypotension, abdominal distension	6 h	HCT 30	Paracentesis	Splenic suture	Alive
1973	5 (6)	34 weeks	2.0	Erythroblastosis fetalis	C/S	NA	Abdominal girth increased	30 h	HCT 14	Paracentesis	Repaired by oxidized cellulose	Died on the second postoperative day
1975	6 (7)	36 weeks	3.08	Erythroblastosis fetalis	VD	4→ 9	Anemia	48 h	Hb 7.2	Paracentesis	Splenectomy	Alive
1980	7 (8)	40 weeks	3.975	NA	VD	9→ 10	Pale, tachypnea, shock	NA	Hb 6.4	Paracentesis	Exploratory laparotomy	Alive
1984	8 (9)	Term	NA	Mother took luminal	VD	NA	Lethargy, pale, and abdominal distension	24 h	HCT 19.6 PLT 850K	X-ray: free fluid	Exploratory laparotomy	Alive; normal
1993	9 (10)	Term	3.05	NA	VD	9→ 10	Scrotal sac dilatation, bilateral hematoceles, hematoma in the left inguinal canal, hematoma of the left anterior abdominal wall	24 h	Normal	US	Exploratory laparotomy	Alive; Normal follow-up examinations at 4 and 6 months of age
1993	10 (10)	Term	3.2	Prolonged labor	VD	7→ 10	Hematoma of the left scrotum and inguinal canal, abdominal distension	16 h	Hb:5.9 HCT 20%	US	Splenic auto-transplantation	Alive; Normal follow-up examinations
1999	11 (11)	36 weeks	2.9	Hemophilia A Preterm	NA	8→ 9	Pale and lethargy, swollen right scrotum	5 days	HCT 6.2%	US	Splenectomy	Alive, discharge on day14
2000	12 (12)	39 weeks	4.167	Prolonged labor	VD	8→ 9	Pale, grunting, lethargy, abdominal distention	2 days	HCT 16% PLT 198K	X-ray: Unremarkable	Splenectomy	Alive; discharged on postoperative day 8
2000	13 (13)	Term	2.82	Birth trauma	VD	8→ 10	Death was noted by nurse	7 h	NA	Autopsy	Non	Dead at 7 h old
2000	14 (14)	26 weeks	0.746	Prolonged labor Preterm Resuscitation	VD	0→ 1	Hypotension, tense and discolored abdomen	36 h	Hb 11.6	US	Packed the site with surgical and 2-component fibrin sealant	Alive
2000	15 (1)	Term	3.6	Prolonged labor	VD	8→ 9	Abdominal distension, pale looking	<5 h	Hb 4.3	CT	Blood transfusion	Alive; discharged at day 10
2002	16 (2)	38 weeks	3.55	NA	VD	8→ 9	Coffee ground emesis, abdominal distension, pale	16 h	Hb 8.5	Paracentesis, US	Packed with gel-foam and surgical	Alive, discharged at day 22
2003	17 (15)	Term	5.08	NA	VD	8→ 9	Right hemiscrotal swelling and bluish discoloration	4 h	Hb 10.6	US, CT	Blood transfusion	Alive
2004	18 (16)	Term	3.6	NA	VD	8→ 9	Bilious vomiting, pale, abdominal distension	2 days	Hb 5.8	CT	Conservative treatment	Alive, discharged at hospital 12
2004	19 (17)	36 weeks	2.62	Wandering spleen Preterm	C/S	8→ 10	Intense mucous and cutaneous paleness, scant motility, hypotonia, ecchymosis in the right side of the scrotum	22 h	Hb 5.5 HCT 16% PLT 155K	US, CT	Peritoneal flap	Alive; normal at the age of 1 year old
2006	20 (18)	27 weeks	0.845	Breech Preterm	VD	6→ 8	Apnea, sudden hypotension, pallor, poor perfusion, abdominal distension	20 h	Hb 6.4	Non	argon beam and tissue glue	Alive; normal at the age of 11 months
2007	21 (19)	36 weeks	2.3	Splenic cavernous hemangioma	VD	NA	Hypovolemia, anemia	48 h	Hb 8.5	US, CT	Splenectomy	Alive
2008	22 (20)	Term	3.2	Non	VD	NA	Progressive pallor, abdominal distension, poor feeding, bruising on the left side of neck, chest and both soles	30 h	HCT 18.9	US, CT	Conservative treatment	Alive; discharged at 15 days of age; resolution of hematoma at 1 month old
2010	23 (21)	Term	3.452	Hemophilia A	VD	9→ 9	lethargy and pale with a weak cry	72 h	Hb 5.0	CT	Splenectomy	Alive
2011	24 (22)	Term	3.64	Assaulted 2 weeks before delivery	VD	NA	Abdominal distension, subconjunctival hemorrhages, facial bruising, pallor, poor feeding	12 h	Hb 9.5 HCT31% PLT 351K	US, Needle paracentesis	Splenectomy	Alive
2011	25 (23)	35 weeks	2.48	Erythroblastosis	C/S	8→ 9	Abdominal distension	3 days	Hb 5.6	Paracentesis, US	Peritoneal drainage, blood transfusion	Alive
2012	26 (24)	Term	3.45	Family history of Hemophilia A	VD	9→ 9	Pallor, lethargy, poor feeding and vomiting	3 days	HCT 14%	CT	Splenectomy	Alive; discharge on POD 31
2015	27 (25)	Term	3.5	NA	VD	NA	Swollen, discolored, and tender left hemi-scrotum	2 days	Hb 7.1	US, CT	Splenectomy	Alive
2016	28 (26)	40 weeks	2.385	Splenic hemangioma	VD	NA	Pale, vomiting, tender abdomen	24 h	NA	US	Splenectomy	Alive
2017	29 (27)	39 weeks	4.2	Congenital deficiency in coagulation factor VII	VD	9→ 10	Pale, grunting, nasal flaring and respiratory distress, extensive parietal cephalohematoma, abdominal enlargement, abdominal tenderness	24 h	Hb 4.0 HCT 14%	US	Splenectomy	Alive; self-limiting coagulation at 1 year old
2017	30 (28)	24 weeks	0.71	Preterm Resuscitation	C/S	6→ 8	Hemoglobin dropped	8 h	Hb 8.7	US, CT	Massive blood transfusion (FFP, LPR)	Alive; resolution of splenic hematoma
2017	31 (29)	40 weeks	2.95	Hemophilia A	VD	Normal	Jaundice and pallor	4 days	Hb 6.9	US, CT	Factor VIII infusions	Alive; discharge at 18 days old; normal growth and development
2017	32 (30)	32 weeks	1.96	Preterm	C/S	3→ 3	Diffuse cutaneous hematomas, bleeding at the puncture points, and bleeding at the umbilical venous catheter	3 h	Hb 4.4	US	Massive blood transfusion	Died at 72 h due to major hyperkalemia with heart rhythm disorders
2017	33 (30)	38 weeks	3.56	Broncho-aspiration and then ventilated with neopuff	VD	3→ 5	Pallor, respiratory distress, abdominal bloating, tachycardia	12 h	Hb 6.4	US	Splenectomy	Alive; Hurler's disease
2017	34 (30)	Term	3.145	NA	VD	7→ 8	Cutaneous-mucosal pallor, respiratory distress, a bloated abdomen and hypotonia	1 h	Hb 10.3	US	Splenectomy	Died at 55 h
2017	35 (30)	Term	NA	Suction extraction	VD	2→ 4	Pallor, shock, abdominal distension	1 h	Hb 10.3	US	Splenectomy	Died at 103 h
2017	36 (30)	40 weeks	2.88	NA	C/S	1→ 6	Pale, abdominal distension	1 h	Hb 10.6	US	Hemostatic mesh	Died at 60 h
2018	37 (31)	Term	3.885	Non	VD	9→ 9	Bluish discoloration, hypotension, marked abdominal distention	6 h	Hb 5.5	US	Blood transfusion	Alive; Normal
Our case	38	Term	2.46	Resuscitation	C/S	6→ 7	Less urine output, hypovolemic shock, abdominal distension	6 h	Hb 8.8 PLT 190K	US	Splenectomy	Dead at 1 month old
Our case	39	39 weeks	3.34	Non	VD	8→ 9	Tachycardia, pale appearance, deteriorating activity	2 days	Hb 5.8 PLT 207K	US, CT	Blood transfusion	Alive; Normal follow-up examinations

*GA, gestational age; BW, birth weight; NA, unknown; VD, vaginal delivery; C/S, cesarean section; AS, Apgar scores; US, ultrasonography; CT, computed tomography; POD, post-operative day; (#) indicates reference number*.

The review of all reported reports revealed that the initial presenting symptoms and signs included pallor or anemia (*n* = 23), abdominal discoloration/distension (*n* = 23), tachycardia/shock (*n* = 10), scrotal hematoma or swollen (*n* = 7), lethargy (*n* = 5), vomiting (*n* = 4), poor feeding (*n* = 3), and death (*n* = 1). Abdominal ultrasonography was performed in 24 neonates; eight of these neonates were also evaluated by abdominal CT as an adjuvant diagnostic tool. Four neonates underwent abdominal CT as the initial diagnostic tool. Twenty-seven neonates (69%) received surgical treatment; 16 neonates received splenectomy; 6 neonates received repair of the spleen rupture with tissue-glue or surgery; 1 neonate received the peritoneal flap; 1 neonate was managed with splenic auto-transplantation; 3 neonates underwent exploratory laparotomy only. There were seven death in this study cohort; therefore the mortality rate was 18% (7/39). Case 13 was found dead at 7 h of age by the nurse; case 32 died at 72 h of age due to severe hyperkalemia with a heart rhythm disorder; case 34 died at 55 h of age; case 35 died at 103 h of age due to massive pulmonary hemorrhage with refractory hypoxemia; case 36 died at 60 h of age, and case 38 died at 1 month of age after withdrawal of life support. None of the remaining 32 survivors (82%) exhibited neurologic sequelae, developmental delays or growth retardation.

Table 2 shows the clinical features associated with mortality. The mortality group had statistically significant higher rates of vaginal delivery (*p* = 0.041, odds ratio = 0.144, 95% CI 0.024–0.853). The age of presentation with neonatal SRH was associated with poor outcome. The odds ratio for mortality was 25.0 (95% CI 2.514–248.575, *p* = 0.001) in neonates presenting with SRH at ≤12 h of age compared to those presenting with SRH >12 h of age. The mortality group had lower age of presentation with neonatal SRH that the median age was 3 h (IQR 1–7). Preterm birth, low BW, prolong labor, birth trauma, resuscitation, 1 min of Apgar score, 5 min of Apgar score, congenital coagulation disorders, hemoglobin level, or splenectomy were not statistically associated with mortality.

**Table 2:** Risk factors associated with mortality in neonates with splenic rupture/hemorrhage.

	**All**	**Survival**	**Mortality**	***p*-value**	**Odds ratio**	**95% CI**
**Categorical variable**						
Patient number (*n*, %)	39	32 (82)	7 (18)			
Preterm (*n*, %)	11	9 (82)	2 (18)	0.981	1.022	0.167–6.258
Prolong labor (*n*, %)	5	4 (80)	1 (20)	0.898	1.167	0.11–12.381
VD (*n*, %)	29	26 (90)	3 (10)	*0.041	0.144	0.024–0.853
Birth trauma (*n*, %)	3	1 (33)	2 (67)	0.077	12.4	0.94–163.581
Resuscitation (*n*, %)	5	3 (60)	2 (40)	0.169	3.867	0.51–29.304
Age of presentation <12 h (*n*, %)	12	6 (50)	6 (50)	*0.001	25.0	2.514–248.575
Splenectomy (*n*, %)	14	11 (79)	3 (21)	0.966	0.964	0.185–5.030
Congenital coagulation disorder (*n*, %)	5	5 (100)	0	0.263	NS	NS
**Continuous variable**						
Age of presentation (IQR)	23 (6–48)	24 (12–24)	3 (1–7)	*0.044	0.879	0.775–0.997
Birth weight (kg) (IQR)	3.2 (2.47–3.62)	3.45 (2.55–3.63)	2.82 (2.0–3.145)	0.193	0.562	0.236–1.338
Apgar score 1 min (IQR)	8 (6–9)	8 (6–9)	4 (2–7)	0.054	0.734	0.536–1.006
Apgar score 5 min (IQR)	9 (8,9)	9 (9)	7 (4–9)	0.114	0.773	0.561–1.064
Hemoglobin (g/dl) (IQR)	6.7 (5.5–8.8)	6.4 (5.5–7.5)	10.3 (6.6–10.5)	0.073	1.583	0.958–2.617

*NS, non-specific*.

## Discussion

We herein presented an uncommon presentation of SRH (case 1) in the cooling era for perinatal HIE. Based on our experience in two cases and the analysis of available literature, presentation at ≤12 h of age was a risk factor for mortality in neonates with SRH. As a rare cause of hemoperitoneum in neonates, splenic injury is less frequently than injury to the liver or adrenal glands due to its well-protected position in the left upper abdominal quadrant (20). The triad of anemia, abdominal distension and shock has been described as typical clinical presentation of intra-abdominal hemorrhage in neonates (1). Clinicians should suspect abdominal hemorrhage if patients had unexplained anemia. Hepatic lesions (65%) account for the most frequent causes of intra-abdominal hemorrhage, followed by adrenal lesions (15%) and splenic lesion (10%) (30). Hepatic laceration should be considered if the patient underwent umbilical venous catheter insertion (32). Patients had history of hypoxia before or during delivery may lead to adrenal gland hemorrhage because the increased pressure with congestion and the damaged endothelial cells (33, 34).

Case 1 neonate presented herein was born to a mother with placental abruption and was diagnosed as perinatal asphyxia with stage II HIE. Therapeutic hypothermia was performed as recommended (35). Abdominal ultrasonography performed based on the observed anemia and progressive abdominal distension revealed splenic rupture with hemorrhage. Case 1 neonate, who was at high mortality risk according to our review and analysis, nonetheless highlights that SRH can be a cause or a comorbid condition in neonates with perinatal HIE undergoing therapeutic hypothermia.

On our review, vaginal delivery was significantly associated with mortality which is thought increased intrathoracic pressure during vaginal delivery pushing the spleen into the abdominal cavity, and exposing it to direct splenic trauma through the birth canal during labor (29). Gruenwald et al. indicated that the intrathoracic pressure that forces the liver or spleen out of the diaphragm hallow can cause severe tension in their supported ligaments (36). Splenic rupture and splenic hemorrhage occur in two stages. The first stage is subcapsular hematoma formation, which is often asymptomatic, whereas the second stage is capsular rupture which leads to the development of acute symptoms of decompensation and the rapid onset of hypovolemic shock (30). The present study revealed that early presentation at ≤12 h of age was associated with poor prognosis. Splenic rupture/hemorrhage in neonates can present either within the first few hours of life or as late as the second week of life, following the rupture of the splenic capsule. In addition, splenic injury can range from the superficial lacerations to the parenchymal hemorrhage. Altogether, early presentation after birth might imply severe and extensive splenic injury, leading to earlier emergence of symptoms present earlier and difficult to recovery.

We also found that the most common presenting symptoms and signs of SRH were pallor or anemia, followed by abdominal discoloration/distension. In the present study, we also noted that 18% (7/39) of all reported cases of neonatal SRH presented with scrotal hematoma or swelling. Most of the affected neonates (74%) were born through vaginal delivery, and four out of them (14%) were LGA neonates. Higher birth weight is not a precipitating factor leading to SRH. Regarding precipitating and risk factors, most cases of neonatal SRH were idiopathic. However, previous studies reported that splenic rupture occurs in those with an abnormal spleen such as those with erythroblastosis (6, 7, 37). Congenital coagulation disorders accounted for 13% (5/39) of SRH of all neonatal SRH cases in the present study cohort. Therefore, investigation for congenital coagulation disorders is recommended in neonates with SRH.

Based on our literature review, the prognosis was comparable between patients who received non-surgical treatment and those who underwent splenectomy. The first case of neonatal SRH who recovered after surgical treatment was reported by Rogers in 1934 (38). Splenectomy has been considered as the first line of treatment for neonatal SRH in the past decade (36), and the preservation of the spleen is desirable (39). Recent studies recommend non-surgical management as the first-line of therapy for SRH due to the risk of overwhelming post splenectomy sepsis (14, 17, 40). Studies also indicated that children are at higher risk of post-splenectomy sepsis compared with adults (40, 41). In addition, advances in ultrasound technology allow the definitive diagnosis of causes underlying intra-abdominal hemorrhage such as renal and splenic hemorrhage (42, 43). Computed tomography to evaluate the severity of splenic trauma may also provide safe non-surgical management in these patients (44). With recent improvement in diagnostic imaging and alternative approaches, splenectomy is no longer a priority. Our literature review reveals that long-term outcome is favorable in neonates who survive SRH.

The present study has several limitations. First, the small sample size of the study might have affected the statistical analysis. However, neonatal SRH is extremely rare and the current study included the largest cohort in statistical analyses. Additionally, the retrospective study designs might have introduced bias.

## Conclusion

The mortality of neonatal splenic rupture/hemorrhage was 18% in this study. Vaginal delivery and the presentation at ≤12 h of age were risk factors for mortality. Investigation for congenital coagulation disorders is recommended in neonate with SRH.

## Data Availability Statement

The raw data supporting the conclusions of this article will be made available by the authors, without undue reservation.

## Ethics Statement

The studies involving human participants were reviewed and approved by the ethics committee of Chang Gung Medical Hospital. Written informed consent for participation was not provided by the participants' legal guardians/next of kin because: this retrospective study was approved by the ethics committee of Chang Gung Medical Hospital and waived the requirement to obtain informed consent of collecting anonymized data.

## Author Contributions

H-PC conceived the study, collected information from the literature, and drafted the manuscript. R-HF and J-JL provided professional guidance and manuscript modification. M-CC conceived the study, provided professional guidance, and manuscript modification. All authors contributed to the article and approved the submitted version.

## Conflict of Interest

The authors declare that the research was conducted in the absence of any commercial or financial relationships that could be construed as a potential conflict of interest.
